# Discovery of Small-Molecule Inhibitors of SARS-CoV-2 Proteins Using a Computational and Experimental Pipeline

**DOI:** 10.3389/fmolb.2021.678701

**Published:** 2021-07-09

**Authors:** Edmond Y. Lau, Oscar A. Negrete, W. F. Drew Bennett, Brian J. Bennion, Monica Borucki, Feliza Bourguet, Aidan Epstein, Magdalena Franco, Brooke Harmon, Stewart He, Derek Jones, Hyojin Kim, Daniel Kirshner, Victoria Lao, Jacky Lo, Kevin McLoughlin, Richard Mosesso, Deepa K. Murugesh, Edwin A. Saada, Brent Segelke, Maxwell A. Stefan, Garrett A. Stevenson, Marisa W. Torres, Dina R. Weilhammer, Sergio Wong, Yue Yang, Adam Zemla, Xiaohua Zhang, Fangqiang Zhu, Jonathan E. Allen, Felice C. Lightstone

**Affiliations:** ^1^ Lawrence Livermore National Laboratory, Physical and Life Sciences Directorate, Biotechnology and Biosciences Division, Livermore, CA, United States; ^2^ Sandia National Laboratory, Department of Biotechnologies and Bioengineering, Livermore, CA, United States; ^3^ Lawrence Livermore National Laboratory, Computing Directorate, Global Security Computing Division, Livermore, CA, United States; ^4^ Sandia National Laboratory, Department Systems Biology, Livermore, CA, United States; ^5^ Lawrence Livermore National Laboratory, Computing Directorate, Center for Applied Scientific Computing, Livermore, CA, United States; ^6^ Lawrence Livermore National Laboratory, Engineering Directorate, Computational Engineering Division, Livermore, CA, United States

**Keywords:** COVID-19, molecular simulations, machine-learning, protein assays, FRET, live virus, main protease, spike protein

## Abstract

A rapid response is necessary to contain emergent biological outbreaks before they can become pandemics. The novel coronavirus (SARS-CoV-2) that causes COVID-19 was first reported in December of 2019 in Wuhan, China and reached most corners of the globe in less than two months. In just over a year since the initial infections, COVID-19 infected almost 100 million people worldwide. Although similar to SARS-CoV and MERS-CoV, SARS-CoV-2 has resisted treatments that are effective against other coronaviruses. Crystal structures of two SARS-CoV-2 proteins, spike protein and main protease, have been reported and can serve as targets for studies in neutralizing this threat. We have employed molecular docking, molecular dynamics simulations, and machine learning to identify from a library of 26 million molecules possible candidate compounds that may attenuate or neutralize the effects of this virus. The viability of selected candidate compounds against SARS-CoV-2 was determined experimentally by biolayer interferometry and FRET-based activity protein assays along with virus-based assays. In the pseudovirus assay, imatinib and lapatinib had IC_50_ values below 10 μM, while candesartan cilexetil had an IC_50_ value of approximately 67 µM against M^pro^ in a FRET-based activity assay. Comparatively, candesartan cilexetil had the highest selectivity index of all compounds tested as its half-maximal cytotoxicity concentration 50 (CC_50_) value was the only one greater than the limit of the assay (>100 μM).

## Introduction

In December 2019, the first cases of a novel coronavirus (SARS-CoV-2) were reported in Wuhan city, Hubei province of China (World Health Organization, 2020). Symptoms of the first patients were flu-like and included fever, dry cough, headache, and myalgia, but with a tendency to develop into potentially fatal dyspnea and acute respiratory distress syndrome (Huang et al., 2020). Within a matter of weeks this coronavirus had spread to many parts of China and preliminary evidence suggests its ability to pass between people without showing outward symptoms (Rothe et al., 2020). Additionally, its transmissibility is higher than that of SARS-CoV (Xia et al., 2020). These features and likely others in the coronavirus as well as the ease of international travel has allowed the outbreak to reach every populated continent. Many countries have taken the extraordinary measure of locking down cities with populations in the millions to slow the spread of the virus. As of this writing, over 98,000,000 people have contracted SARS-CoV-2 with more than 2,100,000 fatalities worldwide (WHO Coronavirus Disease, 2020). Phylogenetic analysis of the genomic sequence of SARS-CoV-2 has shown that it is a member of the betacoronavirus genus and related to SARS-CoV and MERS-CoV (Letko et al., 2020). SARS-CoV-2 has so far has been shown to be resistant to treatments developed for its related viruses although the compound remdesivir has shown some promise and has been approved for emergency use (Beigel et al., 2020).

A concerted effort worldwide has been placed on solving protein structures from SARS-CoV-2 to better understand the lifecycle of the virus and to provide targets for vaccines and drugs (Scudellari, 2020). The trimeric spike protein was the first protein from SARS-CoV-2 to be solved and was shown to be very similar in structure to the homologous protein in SARS-CoV (Wrapp et al., 2020). Coronaviruses utilize the spike protein to recognize binding sites on cells and anchor themselves to invade their host (Belouzard et al., 2012). The spike protein has been solved by X-ray crystallography and cryo-electron microscopy with its receptor binding domain (RBD) in complex with the human receptor protein angiotensin-converting enzyme 2 (ACE2) (Lan et al., 2020; Wrapp et al., 2020). The binding of RBD to human ACE2 that allows the virus to enter the cell is very strong at 4.7–14.7 nM but surprisingly the binding interaction does not occur over a large surface area (Lan et al., 2020; Wrapp et al., 2020). Many of the ACE2-RBD interactions are located within two large loop regions in the RBD and primarily through sidechain-sidechain interactions.

The other solved protein structure from SARS-CoV-2 used in this study is the main protease (Mpro). The Mpro is a cysteine protease with a catalytic dyad consisting of Cys145 and His41. The dimeric main protease is ubiquitous in coronaviruses and plays a pivotal role in viral gene expression and replication through proteolytic processing of replicase polyproteins (Ullrich and Nitsche, 2020). The SARS-CoV-2 Mpro structure has recently been solved with the covalent inhibitor N3 and released in the Protein Data Bank (PDB, 6LU7) (Jin et al., 2020). A second structure of the SARS-CoV-2 Mpro was made available without a bound inhibitor (6Y84) (Owen et al., 2020). The main protease has a large gorge that binds and cleaves polypeptides that are critical for maturation of the virus and is an attractive site for new inhibitors.

The RBD domain of the spike protein and Mpro are promising targets for *in silico* small molecule studies to find molecules with inhibitory properties. We have performed a combined molecular docking, molecular dynamics simulation, and machine learning study in an effort to identify molecules that may bind to the RBD domain and/or Mpro. These bound molecules may attenuate or neutralize the effects of this virus. These predicted ligands were then tested experimentally for their ability to bind their partner protein using biolayer interferometry for the spike protein and a FRET-based reporter substrate for Mpro. Compounds that were found to bind were further tested in virus-based assays to determine their ability to neutralize SARS-CoV-2.

## Materials and Methods

### Molecular Dynamics Simulations of the Apo-Proteins of the RBD of Spike and Main Protease

Classical molecular dynamics simulations were performed using the program OpenMM (Version 7.4) (Eastman et al., 2017). The AMBER force field was used for the proteins in the system (Maier et al., 2015). The individual proteins (RBD of the spike protein or the dimer of the main protease) were solvated in a TIP3P water box (Jorgensen et al., 1983) and the appropriate numbers of ions (Na^+^ or Cl^−^) were added to neutralize the system. M^pro^ was modeled as its biologically-appropriate dimer. AM1-BCC charges (Jakalian et al., 2002) were used to model the thiolate of Cys145 and His41 was modeled as protonated in M^pro^. The density of the water was simulated at 1.0 g/ml. The energy of the system was minimized before dynamics. The molecular dynamics simulations were performed in an NPT ensemble using the Langevin integrator (Salomon-Ferrer et al., 2013b). The system was coupled to a Monte Carlo thermostat at 300 K. Non-bonded interactions were cutoff at 8 Å. The electrostatics was treated using Particle Mesh Ewald summation with an 8 Å real space cutoff and a 1 Å grid (Darden et al., 1993). SHAKE was used to constrain bonds containing hydrogens (Ryckaert et al., 1977). A 2.0 fs timestep was used and each simulation was run to 100 ns. The temperature of the system was increased in increments of 50 K for 100 ps. Positional constraints were placed on backbone atoms (C, N, and CA) with a force constant of 1 kcal/mole•Å^2^ while the temperature was increased. Once the system has reached 300 K, an additional 1.5 ns of dynamics was performed with the positional constraints, after this time period 100 ns of dynamics was performed without the constraints.

### Molecular Docking and Rescoring Calculations

The in-house ConveyorLC toolchain (Zhang et al., 2014; Zhang et al., 2017) was used to automate the docking and rescoring of compounds against each of the four binding sites identified (two spike sites and two M^pro^ structures/conformations). This toolchain comprises four parallel programs for protein preparation (CDT1Receptor), ligand preparation (CDT2Ligand), molecular docking (CDT3Docking), and Molecular Mechanics/Generalized Born-Solvent Accessible Surface Area (MM/GBSA) rescoring (CDT4mmgbsa). The ConveyorLC toolchain depends on a number of external libraries, including the Message Passing Interface (MPI) library, the C++ Boost library, the Conduit library, the HDF5 library, and several molecular simulation packages, including Autodock Vina, (Trott and Olson, 2010) the AMBER molecular simulation package (Salomon-Ferrer et al., 2013a), and MGLTOOLS (Morris et al., 2009). Computational results are aggregated and saved in a series of HDF5 files. A few auxiliary tools are included in the toolchain to query and extract data in the HDF5 files.

Over 26 million compounds were selected from four publicly available compound libraries for docking. The ZINC database (Sterling and Irwin, 2015) FDA-approved and “world-not-FDA” drugs were assembled into a “world-approved 2018” set. From ChEMBL, approximately 1.5 million unique compounds were used (Gaulton et al., 2012). From Emolecules, approximately 18 M compounds were used (eMolecules, 2020). The remaining compounds were selected from the Enamine “REAL” database of over 1.2 billion enumerated structures of drug-like compounds predicted to be synthetically feasible (Enamine, 2020).

The CDT3Docking in the ConveyorLC toolchain is based on Autodock Vina (Version 1.1.2) and uses MPI and a multithreading hybrid parallel scheme (Trott and Olson, 2010; Zhang et al., 2013). The docking grids of the binding sites were determined by the protein preparation program in the toolchain. Compounds were prepared for docking in the following manner. SMILES strings and 2D SDF structures were imported into the Molecular Operating Environment (MOE) [Molecular Operating Environment (MOE), 2020] for removal of salts and metal-containing ligands, protonation states were set to the dominant form at pH 7, 3D structures were created and minimized, and relevant MOE descriptors were calculated. The final structures were exported from MOE as SDF files. These structures were then further processed by the ligand preparation in the toolchain by utilizing antechamber and the GAFF force field from the AMBER simulation package (Salomon-Ferrer et al., 2013a).

The over 26 million compounds described above were individually docked into each binding site for a total of more than 100 million docking simulations. An exhaustiveness of 16 was used for ligand pose sampling. The top 10 poses were kept for each docking calculation. Compounds that had a docking score equal to or better than −7.5 kcal/mole were saved in HDF5 files for further study. Using this score threshold, we selected ∼1% of total compounds or approximately 1 million protein-compound complexes for each binding site.

The selected protein-compound complexes were rescored using CDT4mmgbsa in the ConveyorLC toolchain. A total of ∼10 million poses were rescored for each binding site because each complex typically had 10 docking poses. CDT4mmgbsa employs a master-worker parallel scheme, where the master is in charge of job dispatching and each worker receives jobs from the master and performs an MM/GBSA calculation using the AMBER sander program. The AMBER force field (amberff14SB) (Maier et al., 2015) was used for the proteins; the apo proteins’ MM/GBSA energies were previously determined in the CDT1Receptor step. Partial atomic charges for the compounds were computed by antechamber using the AM1-BCC method (Jakalian et al., 2002); each compound’s charges were previously calculated by the CDT2Ligand step. An energy minimization–1,000 steps of steepest descent and 1,000 additional steps of conjugate gradient–was performed on each docked compound-protein complex using the modified generalized Born model of Onufriev, Bashford, and Case with model 2 radii (igb = 5) (Onufriev et al., 2000) with a nonbonded cutoff of 25 Å. The MM/GBSA energy of the minimized protein-compound complex structure was calculated using an infinite cutoff (999 Å) and a protein dielectric constant of 4. The binding affinity was computed by MM/GBSA energy of the complex subtracted from the sum of the MM/GBSA energies of the apo protein and the isolated compound.

### Molecular Dynamics Simulations of World-Approved 2018 Co-Complexes

Molecular dynamics (MD) simulations were performed for each of the world-approved 2018 complexes down-selected from the top 1% of docked compounds (see Supplementary Table S1). The best scoring single-point MM/GBSA co-complex structure was selected as a starting conformation for the MD simulations. The MD simulations were performed using the pmemd_cuda program in AMBER (Salomon-Ferrer et al., 2013b). The catalytic dyad (His41-Cys145) of the main protease was modeled as charged residues. Charges for the thiolate of Cys145 were obtained from AM1-BCC calculations (Jakalian et al., 2002). The General Amber Force Field (GAFF) was used to model the ligands (Wang et al., 2004). The ligand-protein complex was solvated into a truncated octahedron of TIP3P water (Jorgensen et al., 1983), 50 Na^+^ ions with a neutralizing number of Cl^−^ ions were added to the solution. The system was energy minimized with 500 steps of steepest descents and 1,500 steps of conjugate gradients. Initial equilibration was performed with NVT dynamics at 300 K for 200 ps with positional constraints (K = 1 kcal/mole•Å^2^) on the CA atoms in residues. Electrostatic interactions were treated using Particle Mesh Ewald (PME) summation (Darden et al., 1993). The nonbonded interactions were cut off at 8 Å. Further equilibration was performed with NPT dynamics for 4.8 ns. The pressure was set at 1 atm using a Monte Carlo barostat (Salomon-Ferrer et al., 2013b). The positional constraints were reduced to 0.5 kcal/mole•Å^2^). Production dynamics was performed for 200 ns without positional constraints. The MM/GBSA energies were calculated using MMPBSA.py (Miller et al., 2012) utilizing the Generalized Born model of Onufriev, Bashford, and Case (igb = 5) (Onufriev et al., 2000) on coordinates saved every 20 ps.

### Machine Learning

To assist in determining promising compounds that may have missed the energy cutoff and complement MM/GBSA rescoring, we utilized our Structure-Based Deep Fusion Inference models. We will only briefly describe the Fusion methods, which is described in detail in a previous publication (Jones et al., 2021).

The Deep Fusion models are based on 3D convolutional neural network (3D-CNN) and spatial graph (SG-CNN) models trained independently on ligand-protein co-crystal structure data from PDBBind 2016 (Liu et al., 2017). Two types of fusion models are then built on top of the CNN layers. In the “Mid-Fusion” model, the intermediate CNN features extracted from each model are combined using a series of fully connected layers and then used to predict a ligand-protein binding score. Batch normalization and ReLU-based non-linearities are applied in each fully connected layer. In the “Late-Fusion” model, we combined the constituent CNN models’ predictions rather than their features to produce the final prediction. We used the two fusion models along with the two component CNN models to rank compounds for spike and M^pro^ inhibition.

We used the 3D configurations from the docking calculations in our pipeline as input for our structure-based deep learning methods. Since these models are trained using the protein binding pocket coupled with the ligand, it was necessary to develop a protocol to extract binding pockets from the SARS-CoV-2 proteins. We considered multiple volumes for the bounding box centered on the ligand centroid. We validated our choices by considering correlation (Pearson and Spearman) of the model predictions across bounding box size for all structure-based machine learning methods while additionally considering consensus with the MM/GBSA rescoring method via Pearson and Spearman correlation. Our results showed that given these metrics, the optimal bounding box configuration varied significantly and suggested that the optimal approach would be to combine results across all configurations.

Using these methods, we computed rankings of the SARS-CoV-2 protein inhibitors by scoring each compound for each target for each candidate bounding box. The predictions were then averaged across all bounding boxes to produce the final score for each protein-ligand combination. Then, for each of the models, the compounds were sorted according to predicted activity and ranked in descending order. The sum of the reciprocal rankings was then used to aggregate the rankings across all methods. The top five unique spike protein inhibitors along with the top 25 unique M^pro^ inhibitors were then chosen for experimental validation.

The pharmacokinetic and safety properties of the 26 million compounds used in this study were predicted with the ATOM Modeling PipeLine (AMPL) (Minnich et al., 2020), a data-driven pipeline for drug discovery, and the Maestro workflow manager (Di Natale, 2017). Chemical descriptors were computed with MOE and Mordred from 2D and 3D structures and graph (Ramsundar et al., 2019) and fingerprint representations. Fully connected neural networks, graph convolution, and random forest models were considered, and the best models selected using AMPL. A total of 30 models with 23 distinct targets were used for property prediction and are summarized in Supplementary Table S2. Results for the 9 models trained on public data are available at https://covid19drugscreen.llnl.gov.

### Spike RBD and ACE2-Fc Protein Production and Purification

The gene for the SARS-CoV-2 spike protein (NC_045512.2) was codon-optimized for expression in mammalian cells and subcloned into pcDNA3.4 with the native secretion signal and a C-terminal His_8_ tag. The plasmid was transfected into Expi293 cells and cultured for 5 days according to the manufacturer (ThermoFisher Scientific). Cells were harvested by centrifugation and the spike-containing culture medium was sterile-filtered, pH adjusted to 7.4 using PBS, and captured on a HisTrap Excel (Cytiva) using the Akta Pure FPLC system. The column was washed with wash buffer (20 mM sodium phosphate, 300 mM sodium chloride, 40 mM imidazole, pH 7.4) and eluted with wash buffer containing 500 mM imidazole. Fractions containing spike RBD were pooled and concentrated using a 10 kDa MWCO centrifugal concentrator (ThermoFisher). The concentrated protein was loaded onto a Superdex 200 Increase 10/300 GL equilibrated with PBS, pH 7.4. Fractions containing spike RBD were pooled and concentrated as before.

The ACE2-Fc fusion construct was made by subcloning the ectodomain of the human ACE2 gene (Sino Biological) into the pCR3-Fc vector, which contains the CH2 and CH3 domains of human IgG1 as previously described (Negrete et al., 2006). The ACE2-Fc containing plasmid was transfected into ExpiCHO cells and cultured for 7 days according to the manufacturer (ThermoFisher Scientific). Cells were harvested by centrifugation and the ACE2-Fc-containing culture medium was sterile-filtered, pH adjusted to 7.4 using PBS, and captured on a MabSelect PrismA column (Cytiva) using the Akta Pure FPLC system. The column was washed with wash buffer (50 mM sodium phosphate, 150 mM sodium chloride, pH 7.4) and eluted with 100 mM sodium citrate pH 3. Fractions containing ACE2-Fc were pooled and concentrated using a 10 MWCO centrifugal concentrator (ThermoFisher). The concentrated protein was loaded onto a Superdex 200 Increase 10/300 GL equilibrated with PBS, pH 7.4. Fractions containing ACE2-Fc were pooled and concentrated as before.

### Biolayer Interferometry Competition Assay for Spike Protein binding Compound

The competitive binding assays were performed by biolayer interferometry using the Octet RED96 system (FortéBio). All experiments were performed using 96 well microplates (Greiner Bio-One) at 30°C with the shaking speed of 1,000 rpm and samples were diluted in kinetic buffer (PBS containing 0.02% Tween 20, 0.1% bovine serum albumin). Octet anti-human Fc (AHC) biosensors were pre-equilibrated in biosensor buffer [kinetic buffer (KB) containing 10 µg/ml biocytin] for 30 min before use in experiments. SARS-CoV-2 RBD was pretreated with candidate compounds for 30 min prior to assay start. Human ACE2-Fc protein was immobilized on the surface of the AHC biosensor tip and followed by a baseline step of 120 s in KB. ACE2-captured biosensors were immersed in wells containing different concentrations (5–100 µM) of small molecule and SARS-CoV-2 RBD for 180 s followed by dissociation step for 200 s. The raw data was analyzed using Octet Data Analysis High Throughput software (FortéBio). Binding sensorgrams were aligned at the beginning of the binding cycle, double reference subtracted and Savitzky Golay filtered data were globally fit to a 1:1 binding model. A total of 32 compounds (see Supplementary Table S3) were tested against the RBD. All compounds were purchased from TargetMol at 97% purity or higher and used without further purification.

### M^pro^ and FRET Substrate Protein Production and Purification

The gene for the SARS-Cov-2 M^pro^ (from Genbank MN908947.3) was codon-optimized for expression in *E. coli* and subcloned into a pET-32 vector, with a N-terminal GST tag connected by an auto-cleavage sequence and a C-terminal His_6_ tag. The plasmid was transformed into BL21 DE3 *E. coli* and streaked onto ampicillin plates. Individual colonies were picked and used to inoculate 50 ml starter cultures, which were grown in lysogeny broth (LB) containing ampicillin overnight at 37°C. The 50 ml starter cultures were then used to inoculate 1 L of LB, which was incubated at 37°C until OD = 0.6 to 0.9, at which point IPTG was added to a final concentration of 400 µM and cells were incubated with gentle shaking at 16°C overnight. Cells were then pelleted, flash frozen in liquid nitrogen, and stored at −80°C. The pellet from 100 ml of culture was thawed, resuspended in 10 ml BugBuster master mix (Millipore Sigma), and gently inverted at 4°C for 1 h to lyse. The insoluble fraction of the lysate was then spun down and the supernatant was sterile-filtered prior to capture on a Ni NTA column. The lysate was diluted with Buffer A (20 mM Tris, 100 mM NaCl, 5 mM Bme, pH 8.0), and Ni NTA Buffer B (20 mM Tris, 100 mM NaCl, 5 mM Bme, 500 mM imidazole, pH 8.0) was added to a final concentration of 10 mM imidazole. The lysate was then loaded onto a 5 ml HisTrap Ni NTA column (GE Healthcare) using an FPLC system (Bio-Rad), and eluted with Ni NTA Buffer B. Fractions containing the eluted protein were pooled and spin-exchanged into Buffer A using 10 kDa MWCO Amicon Ultra centrifugal filters (Millipore Sigma). The C-terminal His_6_ tag was then cleaved off by incubating the concentrated protein with 30 µg of N-terminally His-tagged HRV-3C protease (Sigma-Aldrich) overnight at 4°C. The digested protein was applied again to the Ni NTA column, and the flowthrough was collected and used directly for ion exchange chromatography.

The flowthrough was loaded onto a 5 ml High Q anion exchange column (Bio-Rad) and proteins were eluted with a linear gradient of IEX Buffer B (20 mM Tris, 1 M NaCl, 5 mM Bme, pH 8.0). To our surprise, the M^pro^ was found in the flowthrough rather than the eluted fractions. The flowthrough was collected, buffer exchanged into storage buffer (20 mM Tris, 150 mM NaCl, 1 mM TCEP, pH 7.8), flash-frozen, and stored at −80°C. Purity appeared to be >99% by SDS-PAGE and staining with SimplyBlue SafeStain (ThermoFisher).

The fluorescence resonance energy transfer (FRET)-based M^pro^ substrate was cloned into pET bacterial expression vector starting from a pcDNA.31-Clover-mRuby2 plasmid with a cloned linker sequence FGAAR**AVLQSGFR**AADP between the Clover and mRuby2 FRET protein pairs. The cloned linker sequence is a protease substrate and cleaves the peptide backbone between residues **QS**. pcDNA3.1-Clover-mRuby2 was a gift from Kurt Beam (Addgene plasmid # 49089; http://addgene.org/49089; RRID:Addgene_49089). The kanamycin-resistant pET plasmid was transformed into BL21(DE3) cells (NEB) and cultures were induced with IPTG (0.5 mM) at 15°C overnight with gentle shaking (150 RPM). The FRET substrate was subsequently purified by standard Ni-NTA affinity techniques, as described above.

### M^pro^ FRET-Based Activity Assay

M^pro^ inhibitor screening and half maximal inhibitory concentration (IC_50_) analysis were performed in 384 well assay plates, in 25 ml final volumes using 1875 ng of substrate and 375 ng of M^pro^ diluted in assay buffer (0.0033% Triton-X100, 50 mM Tris-HCl, 150 mM NaCl, pH 7.4). All compounds were diluted with DMSO to volumes of 2.5 μl to obtain a 10% final concentration of DMSO in the 25 μl reaction. Percent cleavage of the FRET substrate was measured on a Tecan Spark^®^. Fluorescence emission at 620 nm was measured for each well using excitations at 560 nm (excite mRuby2, emit mRuby2), and 485 nm (FRET from Clover to mRuby2). The FRET signal was normalized to the fluorescence of mRuby2 for each well. All assays were run in technical replicates and averaged. This data was then normalized to the average of the -protease wells (16 replicates per plate). The data was then analyzed in GraphPad Prism 9, wherein the “Normalize” tool was used to set the %FRET values for the +protease control to 0 and the–protease controls to 1.0. Both protease controls utilized 16 replicates per plate. The Z-factor is calculated using the + and -protease control wells (Zhang et al., 1999). This sets the min/max signals for normalization. All wells had DMSO, as compounds were in DMSO. Complete reactions were run on SDS-PAGE gels to assess protein cleavage independently of FRET measurements. Gel densitometry analysis (analyzed using ImageJ) justified the 100 and 0% cleavage in the +protease and –protease controls, respectively, at the time points used for analysis. In each experiment, measurements were taken at several time points, however only end-point data (at which time the +protease control reactions have gone to completion) has been presented herein, at about 4 h post addition of protease. A total of 91 compounds (see Supplementary Table S4) were tested against M^pro^. All compounds were purchased from TargetMol at 97% purity or higher and used without further purification.

### Viral Infection Assays

A pseudotyped, replication-competent vesicular stomatitis virus (VSV) expressing the SARS-CoV-2 spike gene (VSV-SARS2) in place of its own VSV-G gene was provided by Dr. Sean Whelan (Case et al., 2020) and used to screen compounds predicted to target the SARS-CoV-2 spike. VSV-SARS2 also expresses GFP allowing for rapid analysis of infection based on reporter expression under BSL-2 containment. Initial drug screening was performed by incubating the compounds at 10 µM with VSV-SARS2 or VSV-GFP (VSV) as a specificity control for 30 min prior to their addition to Vero cells seeded in a 96-well plate. Infection was performed for 1 h with a multiplicity of infection (MOI) of 0.5 for VSV-SARS2 and 0.1 for VSV. The infection media was subsequently removed, replaced with fresh media and fluorescent protein measurements were collected 18–24 h post-infection. Down-selected compounds were subjected to IC_50_ analysis using dilutions of drug starting at 100 mM concentrations following a similar infection protocol against VSV-SARS2 under BSL-2 containment or recombinant SARS-CoV-2 expressing the mNeon reporter gene (provided by Dr. Pei Yong Shi) (Xie et al., 2020) under BSL-3 containment. The compounds were screened starting at 100 μM using an 8-point, 1:2 dilution series with infections being performed at a MOI of 0.2. In addition, Presto-Blue cytotoxicity assays were performed using a similar dilution series in uninfected cells to determine relative cell viability to drug-only treatments. Fluorescent values were background subtracted using no-infection controls and normalized to no-treatment infection values. IC_50 _curves and values generated using GraphPad Prism 9.

## Results and Discussions

### Computational Predictions

Molecular dynamics simulations were performed on both the RBD of the spike protein and the M^pro^ to identify alternative conformations from the crystal structure (PDB, 6M0J) (Lan et al., 2020). For the RBD structure, a total of twelve 100 ns simulations were performed (aggregate 1.2 µs of dynamics). The structures from the last 20 ns from each simulation was collected and clustered. There were only slight changes in the conformation of residues that would form interactions with ACE2. The most variable region within RBD was located at the opposite end of the protein relative to the ACE2 binding sites. The stability of the ACE2 binding regions likely is not surprising given the high binding constant of RBD to ACE2 and relatively small contact region (Lan et al., 2020). The dynamics of the M^pro^ dimer shows the residues near the active site are stable except for the loop formed by residues Cys44-Pro52 (Bzowka et al., 2020). This loop shifts position in both monomers and moves the associated residues further from the active site.

**FIGURE 1:**
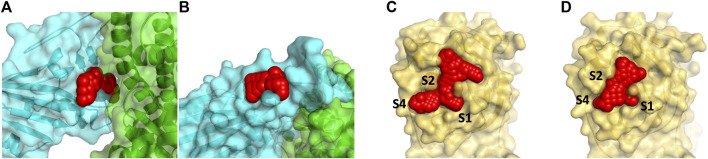
Panel **(A)** shows the docking site on the RBD of the spike protein in red (by residues 501–505) that are at the interface with ACE2 (show in green) and denoted spike1 in the text. A smaller secondary binding site (denoted spike2) in the spike protein in receptor binding motif domain was detected and used for docking studies **(B)**. Panel **(C)** and **(D)** show the binding site of the M^pro^ with the N3 inhibitor removed (6LU7) is protease1 and the apo protein (6Y84) is protease2. The S2 binding pocket is below the sidechains of Met49 and Gln189 and is not visible in the picture.

We identified two binding sites within the RBD of the spike protein and within the M^pro^ proteins binding sites as shown in Figure 1. In the RBD, two sites were chosen that are proximal to critical residues that bind human ACE2. Both sites in the RBD are formed by stable loop areas. The first site is in the proximity of a beta-turn formed by residues 501–505 and denoted spike1 below. This region forms several interactions with ACE2 and the corresponding residues in the SARS-CoV-2 spike protein form the major recognition site for neutralizing antibodies. We used the crystal structure (PDB, 6M0J) for docking to this site since the protein conformations sampled from MD simulations did not significantly differ from the crystal structure. The second site is stabilized by a disulfide (Cys480-Cys488) that connects the loop at the end of the receptor-binding motif (RBM) and denoted spike2 below. These two regions include the two key mutations of the variants of concern–E484K and N501Y (Voloch et al., 2020; Fiorentini et al., 2021). During the MD simulations, it was observed that the sidechains of residues Lys458 and Glu471 become solvent-exposed. In the crystal structure, these two residues are in close proximity and divide a potential binding site into two small sites. In the MD structure, these residues are solvent-exposed and a single larger binding site is present (Figure 1B). We used the MD structure for docking to this site. We limited our drug discovery efforts on the spike protein to two sites in the proximity of the RBD-ACE2 interface where the small molecule would directly interfere with formation of the protein complex. There are likely other drug binding sites within the spike protein that can affect ACE2 binding (Olotu et al., 2020; Verkhiver, 2020) but determining their locations experimentally is non-trivial.

The main protease is a cysteine protease with a catalytic dyad consisting of Cys145-His41. To accommodate its natural polypeptide substrates, a large gorge is present on the surface of the enzyme. The covalent inhibitor N3 is based on the tripeptide Ala-Val-Leu and reacts with the thiolate of Cys145. Two crystal structures of M^pro^ have been solved recently. The 6LU7 crystal structure was solved with the covalent inhibitor N3 in the active site (denoted protease1 in the text) (Jin et al., 2020). A second structure 6Y84 (denoted protease2 in the text) was solved as an apo protein in a different space group relative to 6LU7 (Owen et al., 2020). This crystal structure’s active site differs from 6LU7 with N3 removed. The sidechains of Met49 and Met165 change positions depending upon having N3 present. The shifts in positions of these methionine residues enlarge the active site. In the MD simulations, Met49 shifts position away from the active site to also enlarge this region. We chose to use the crystal structure of 6Y84 as another site for docking since the changes relative to 6LU7 are small but the positional change in Met49 changes/enlarges the active site. In Figures 1C,D we show the two conformations of the active site, one from each of these crystal structures of M^pro^, were used for our docking study.

We docked over 26 million compounds to these four sites (two spike sites and two M^pro^ structures/conformations) to find possible binders that could either interfere with protein binding (RBD of spike protein) or inhibit substrate binding (M^pro^). Although all the compounds docked to these four sites are supposed to be commercially available or can be synthesized, to expedite experimental testing we will focus our discussion on the world-approved 2018 set. The computational results on the other 26 million compounds docked to the four sites are freely available online at https://covid19drugscreen.llnl.gov. The docking score energy cutoff of–7.5 kcal/mole reduced the number of compounds to 136 in the spike1 site and 50 in the spike2 site in the RBD of the spike protein. The larger binding site of the main protease had a greater number of ligands for further testing, 916 for the protease1 site of the main protease2 site. All these compounds were interrogated for activity using our ML Fusion model and MM/GBSA single point calculations to identify the most promising compounds. Each compound bound to its respective site was ranked from highest to lowest by energies for Vina docking score, MM/GBSA energy, and Fusion model. The final ranking of the compounds in their respective sites were inverted (i.e., 1/rank) and weighted by 1.2•(MM/GBSA) + 1.0•(Fusion model) + 0.8•(Vina docking). We believe the physics-based MM/GBSA to be our most accurate method and molecular docking the least predictive method relative to experiment. Because of the modest number of compounds remaining after the energy cutoff, molecular dynamics simulations were performed on all the ligand-protein complexes to obtain an average MM/GBSA energy and to investigate whether the protein dynamics were altered by formation of the complex.

Disruption of RBD binding to ACE2 would prevent infection by SARS-CoV-2. Docking to the spike1 site on the RBD puts the ligand in direct conflict with ACE2 binding when the protein complex is formed. A relatively small number of compounds were able to make the MM/GBSA rescoring energy cutoff for further molecular dynamics simulations since this binding site is relatively shallow. 134 compounds were simulated in the spike1 site using their five lowest-energy docking poses and their average MM/GBSA was determined from the ligand-protein conformations from the MD trajectory. The root mean squared deviation (RMSD) of the protein backbone from the crystal structure was used as an additional criterion to determine the stability of the ligand-protein complex. To successfully disrupt formation of the protein complex, the compounds must have a low MM/GBSA binding energy and be stable within the binding site. Twenty-eight compounds had an average MM/GBSA below −30 kcal/mole and an RMSD 4 Å or less (recentering and was only performed on the protein) for at least one of their simulations. Some compounds on this list that are of additional interest additional interest are accolate, tasosartan, and olmesartan medoxmil. Accolate is used to control and prevent symptoms of asthma such as wheezing and shortness of breath. Tasosartan is an angiotensin II receptor agonist. Olemsartan medoxomil is an angiotensin II receptor blocker. Several studies have pointed to improved outcomes when COVID19 patients have used angiotensin II receptor blockers/inhibitors (Meng et al., 2020; Sanchis-Gomar et al., 2020; Zhang et al., 2020).

The spike2 binding site is located in the receptor binding motif (RBM) of the RBD. This binding site does not directly interfere with formation of a protein-protein complex, however it is in close proximity with a group of aromatic residues (Phe456, Tyr473, and Tyr489) that form interactions with ACE2. We speculated that having a bound compound proximal to these residues might disrupt the positioning of these aromatic residues and affect ACE2 binding. From an initial 134 compounds, only 50 compounds had a MM/GBSA below −30 kcal/mole and an RMSD less than 4 Å during at least one of the simulations. Interestingly, several of the best binding compounds are diuretics or metabolites (glucuronides). The considerable number of polar and charged residues in the vicinity makes this a favorable environment for the glucuronic acid.

In docking calculations of the main protease, two different crystal structures were utilized for docking because the sidechain positions of Met49 and Met165 in the active site vary due to one structure had the ligand N3 (6LU7) present while the other was empty (6Y84). Although the shape of the active site differs, there were 535 compounds that were common to both structures out of the more than 900 compounds that made the initial −7.5 kcal/mole single point energy cutoff for each protein structure. Since there is no indication which structure is preferred, the compounds were ranked by the sum of their average MM/GBSA energies. Several of the top-scoring compounds that bind to both active site conformations are described here. Cefoperazone is a semi-synthetic beta-lactam antibiotic. Irinotecan is a plant alkaloid that acts as a topoisomerase inhibitor used to treat colon and small-cell lung cancers. Its relatively rigid structure allows it to span the length of the active site. Rutin is a citrus flavonoid consisting of quercetin and the disaccharide rutinose and used as an alternative medicine. Several compounds are protease inhibitors or metabolites of drugs. AFN911 is a metabolite of imatinib (benzylic hydroxylation). Losartan n2-glucuronide is the metabolite of losartan (an angiotensin II receptor antagonist). Saquinavir is an antiretroviral drug (protease inhibitor) used to treat HIV/AIDS. Teniposide is a topoisomerase II inhibitor used for treatment of several childhood cancers. Cabozantinib is a tyrosine kinase inhibitor that is used as mediation for medullary thyroid cancer and renal cell carcinoma. Intriguingly, the angiotensin II receptor blocker olmesartan medoxomil was also predicted to bind well to the spike protein. The compounds rutin, losartan, imatinib, saquinavir, and tenposide have been seen in other computational screens (Bello et al., 2020; Huynh et al., 2020; Pant et al., 2020; Nejat and Sadt, 2021). Losartan and imatinib have undergone clinical trials with COVID19 patients (Aman et al., 2021; Puskarich et al., 2021). Most of the metabolites found in the computational screens unfortunately were not available for purchase.

### Experimental Validation

**FIGURE 2:**
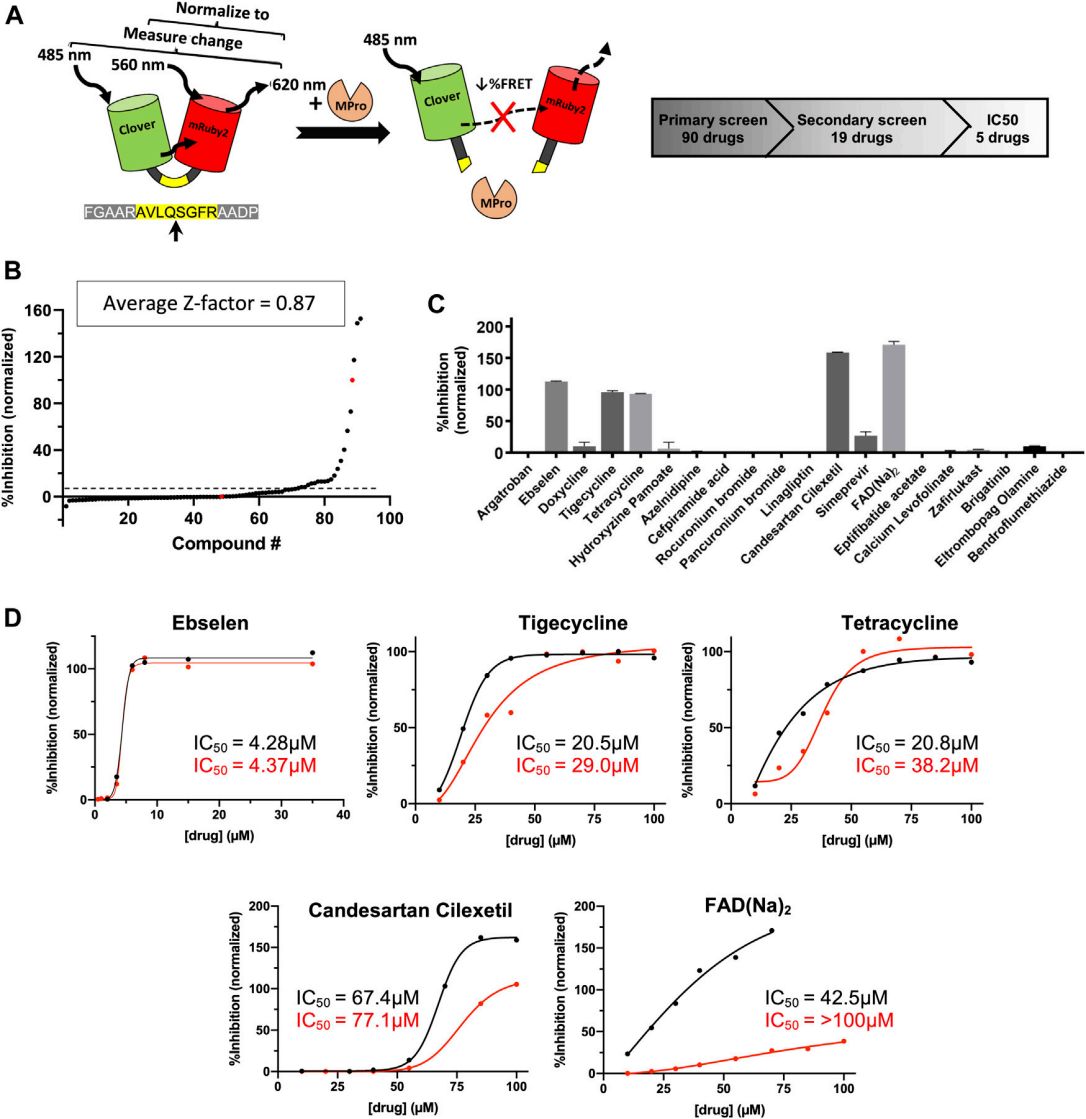
Predicted M^pro^ drug inhibitors screened using a FRET-based protease assay with five down-selected hits. **(A)** A schematic of the FRET-based SARS-CoV-2 main protease assay is shown along with the hit identification overview. **(B)** Purified M^pro^ and FRET substrate proteins were incubated in the presence of 100 μM of drugs from a library of computationally predicted M^pro^ inhibitors. No drug, no protease, and Ebselen were used as controls to calculate the Z-factor for each plate and an average score is displayed above. Red dots indicated no drug (0% inhibition) or no protease (100% inhibition) conditions, while the black dots are the ordered percent inhibition values. **(C)** Identified hits from the primary screen were re-screened at 100 μM and the FRET values were normalized as percent inhibition values in the bar graph. Experiments were performed in duplicate and the presented results are the average values. **(D)** Verified compounds form rescreening were subjected to half-maximal inhibitory concentration (IC_50_) analysis. Presented values are averaged from technical duplicate experiments. Black lines and values represent normalized data from FRET values while the red lines and values represent normalized data from gel electrophoresis (Supplementary Figure S1) and densitometry.

**FIGURE 3:**
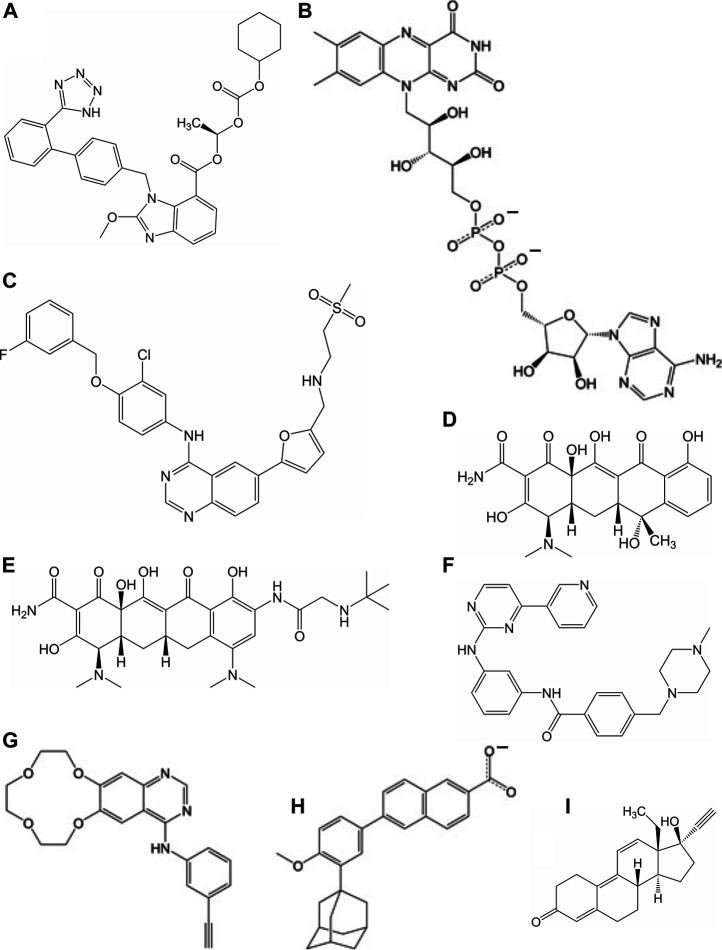
Molecular of structures for compounds that have a repressive effect on some aspect of the virus activity: **(A)** candesartan cilexetil, **(B)** flavin adenosine dinucleotide, **(C)** lapatinib, **(D)** tetracycline, **(E)** tigecycline, **(F)** imatinib, **(G)** icotinib, **(H)** adapalene, and **(I)** gestrinone.

Experimental testing of the predicted binders for Mpro was performed by utilizing a fluorescence resonance energy transfer (FRET) based activity assay (Figure 2A). This FRET assay consisted of a substrate composed of two fluorescent proteins, Clover and mRuby2, linked through a Mpro recognition sequence. Supplementary Figure S1 shows the advantage of a protein-based substrate over standard peptide-based methods was to allow for verification of FRET values by independent, FRET-independent gel electrophoresis. The assay was optimized using a positive control compound called Ebselen, a low micromolar Mpro inhibitor (Jin et al., 2020). Supplementary Table S4 shows the results from our initial screen, from which, 19 compounds were down-selected and tested in a secondary screen where four compounds were found to completely inhibit the activity of Mpro at 100 μM concentrations and are shown in Figure 2C. These identified compounds included candesartan cilexetil, FAD, tigecycline and tetracycline (see Figure 3). Candesartan cilexetil is an angiotensin II receptor antagonist prodrug. Flavin adenine dinucleotide is a redox-active coenzyme. Tigecycline is a glycylcycline antibiotic and closely related to tetracycline. These were the only two compounds that bind Mpro and had a similar molecular structure. In Figure 2D we show that these four compounds were relatively weak inhibitors of Mpro compared to Ebselen as the IC50 values were calculated to be 67.4 µM for candesartan cilexetil, 42.5 µM for FAD disodium, 21.5 µM for tigecycline, and 20.8 µM for tetracycline. The IC50 values were comparable to gel electrophoresis-based analysis of the cleaved substrate products with the exception of FAD as shown in Figure 2D and Supplementary Figure S1. Importantly, candesartan cilextetil has been previously identified as a Mpro inhibitor with a IC50 of 2.8 µM (Li et al., 2020) although the fluorogenic substrate used was slightly shorter than the substrate utilized in this study. Additionally, candesartan cilexetil has inherent fluorescent properties that make determining its cleavage inhibition difficult.

**FIGURE 4:**
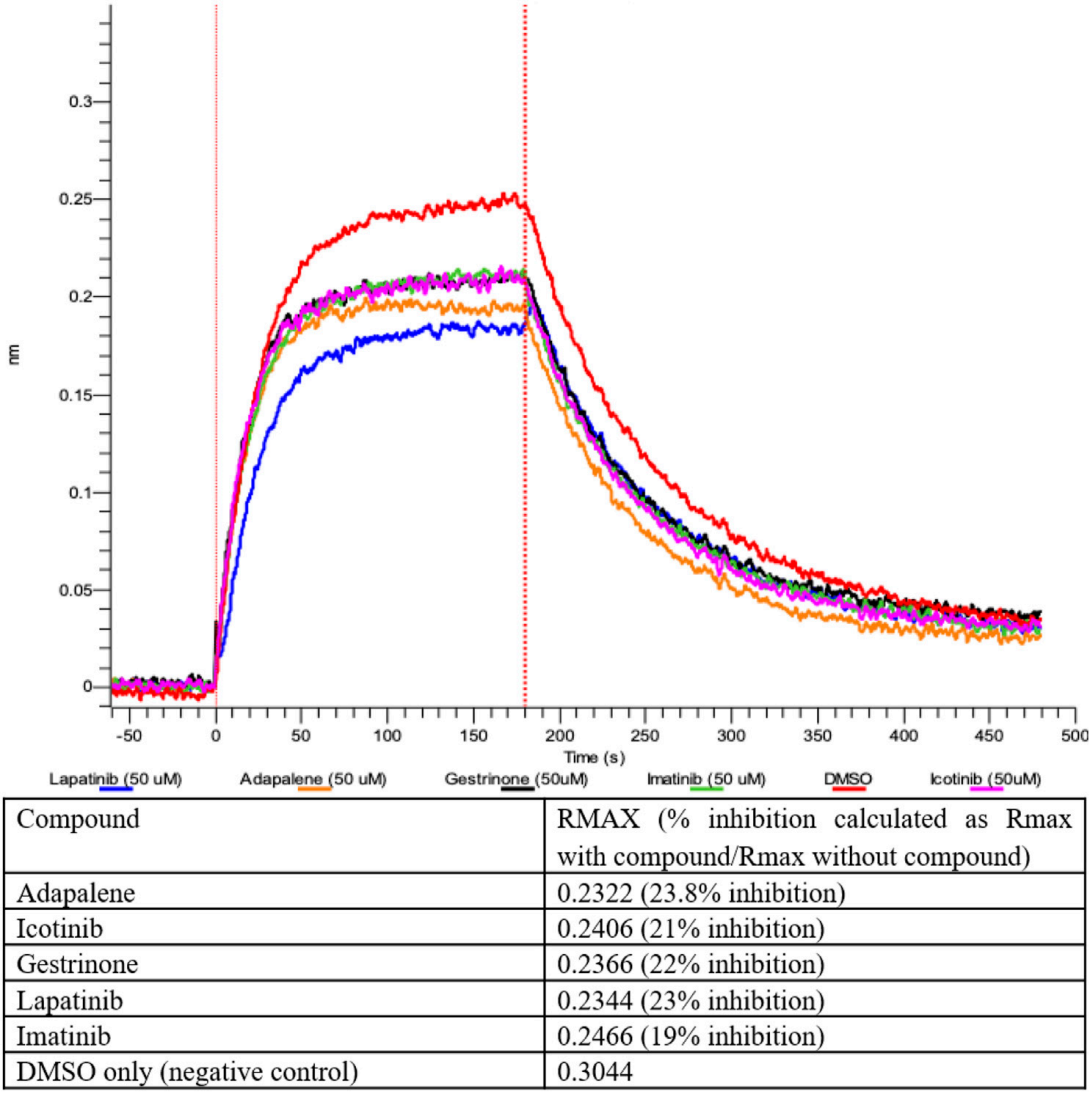
Inhibition of ACE2-RBD binding after pre-treatment with 50 μM compound measured by Biolayer interferometry.

The compounds computationally predicted to target the SARS-CoV-2 spike protein RBD were screened by pseudotyped virus assay and biolayer interferometry competitive assay (BLI). Compounds were tested for their ability to inhibit ACE2-spike binding via BLI competitive assays on Octet RED96 platform (Forte Bio). In this assay, human ACE2-Fc was immobilized on AHC biosensors and binding to soluble SARS-CoV-2 RBD was detected. The RBD was pre-treated with candidate compounds at increasing concentrations prior to assay. In Figure 4, we show an inhibitor concentration-dependent decrease in ACE2-RBD binding in samples pretreated with adapalene, imatinib, lapatinib, gestrinone, and icotinib. Adapalene is a topical retinoid used to treat acne. Icotinib and lapatinib are inhibitors of the tyrosine kinase EGFR. Imatinib is used to treat chronic myelogenous leukemia (CML). Gestrinone is a synthetic steroid used to treat endometriosis. An imatinib metabolite (AFN911) has previously been identified in this study as also a possible Mpro inhibitor.

**FIGURE 5:**
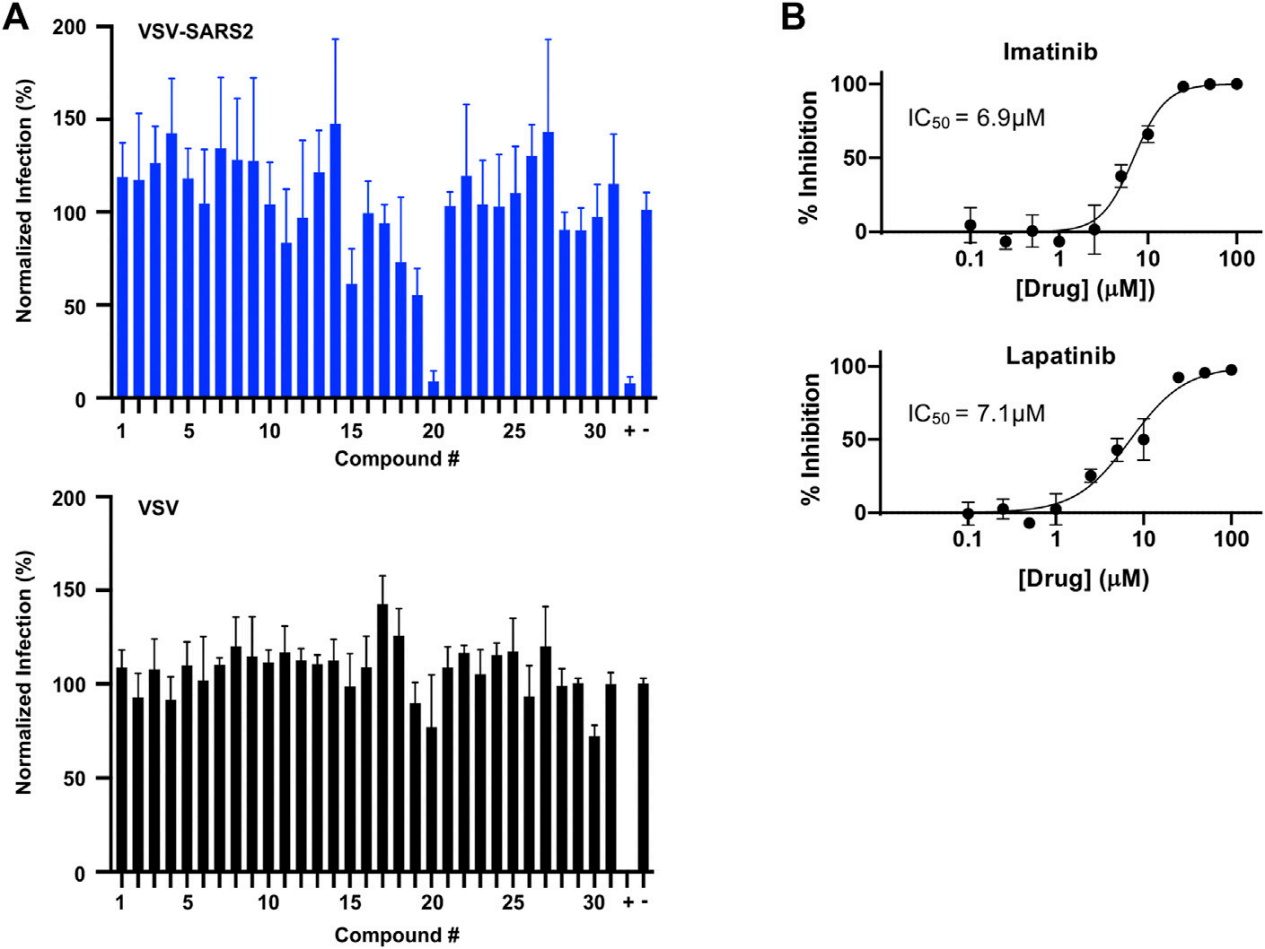
Predicted spike drug inhibitors screened using a VSV-SARS2 infection assay reveals two promising hits. **(A)** Individual drugs from the library set were used at 10 μM to treat GFP reporter viruses, VSV-SARS2 and VSV, for 30 min prior to infection of Vero cells at 0.5 MOI or 0.1 MOI respectively. The infection media was replaced with fresh media at 1 h post-infection and fluorescent reporter values were measured the next day. **(B)** Half-maximal inhibitory concentration (IC_50_) curves and values were obtained for Imatinib (compound 20) and Lapatinib (compound 19) using the same VSV-SARS infection assay performed for library screening. All data were normalized as percent infection or inhibition for drug-treated conditions vs. no-treatment control. The values are means, with error bars displaying standard deviation between the triplicate wells.

In parallel, the computationally-predicted spike binding compounds were screened using a cell-based infection assay. The spike compound library set was screened against a BSL-2 surrogate virus encoding the SARS-CoV-2 spike protein that mimics ACE2-dependent SARS-CoV-2 fusion and cell entry (Case et al., 2020). The replication-competent pseudotyped virus, termed VSV-SARS2 (see Methods), expresses a GFP reporter upon cell infection and replication that was used as an indicator of infection in the drug screen. From the initial library set of 32 compounds, only imatinib and lapatinib were found to inhibit VSV-SARS2 at 10 μM at ∼50% or greater efficacy as shown in Figure 5A and Supplementary Table S3. To check for specificity, the compounds were screened against VSV and none were found to have a significant impact on infection thus indicating the two hits were spike-dependent. The IC50 values of imatinib and lapatinib were 6.9 and 7.1 μM against VSV-SARS2, respectively (Figure 5B).

**FIGURE 6:**
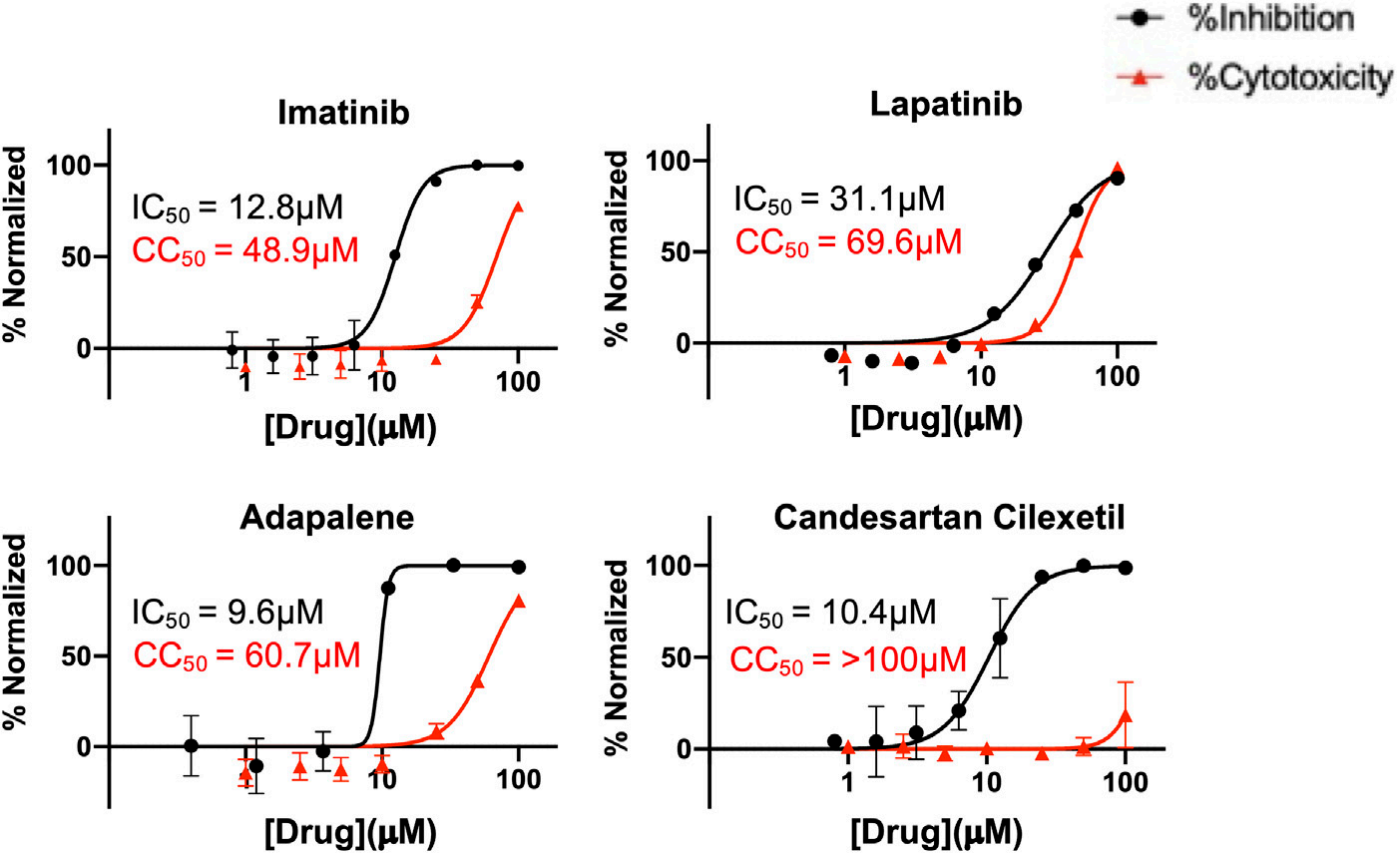
Percentage inhibition and percentage cytotoxicity graphs form SARS-CoV-2 infection studies that show large therapeutic indexes in three hits. Varying concentrations of imatinib, lapatinib, and adapalene were used to treat virus for 30 min prior to infection in Vero cells, while candesartan cilexetil was added directly to cells without pre-treatment to virus. Infections were performed using SARS CoV-2mNeon at an MOI of 0.2. At 1 h post-infection, the media was removed and replaced with fresh media. Fluorescent reporter values were recorded 18 h post-infection. Similarly, Vero cells were treated with varying concentrations of indicated drugs, incubated for 18 h prior to analysis by Presto-Blue assays to assess cytopathic effect. Data were normalized to percent inhibition or percent cytotoxicity for drug-treated cells vs. no-treatment control. The values are means, with error bars displaying standard deviation between the triplicate wells. Half-maximal inhibitory concentration (IC_50_) curves and values are represented in black while half-maximal cytotoxicity concentration 50 (CC_50_) curves and values are represented in red.

**FIGURE 7:**
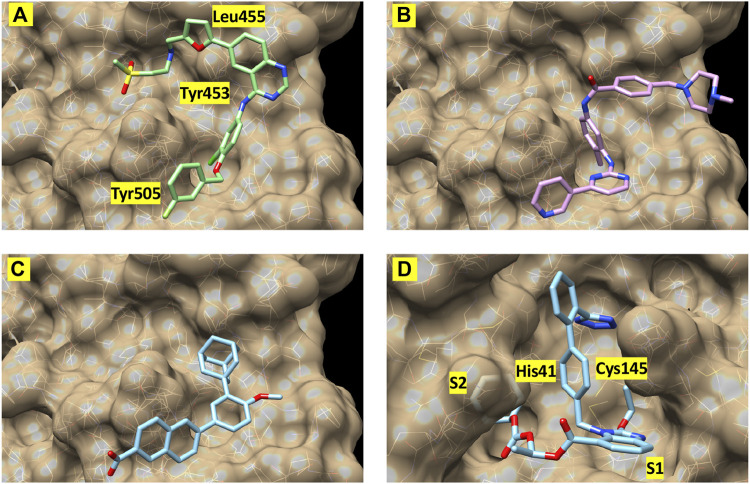
Best-scoring pose from docking for **(A)** lapatinib, **(B)** imatinib, and **(C)** adapalene to the receptor binding domain of the spike protein (spike1 site). Panel **(D)** shows the best-scoring dock pose for candesartan cilexetil to M^pro^. Labels identify protein residues neighboring the docked compounds.

Finally, to further validate the anti-viral effects of identified Mpro and spike hits, the compounds were evaluated under BSL-3 containment using a SARS-CoV-2 reporter virus expressing mNeon (Xie et al., 2020). Figure 6 shows the plotted IC50 and half-maximal cytotoxicity concentration 50 (CC50) graphs for four compounds where virus inhibition was not simply due to the cytotoxicity induced by the drug alone. Imatinib, adapalene and candesartan cilexetil had IC50 values of approximately 10 μM against SARS-CoV-2 in a cell-based assay, while lapatinib had an IC50 value of 31.1 µM. The best scoring conformation of these four compounds with their target protein is shown in Figure 7. Comparatively, candesartan cilexetil had the highest selectivity index of all four compounds as its CC50 value was the only one greater than the limit of the assay (>100 μM, Figure 6). Similar results for candesartan cilexetil were obtained against Vero-E6 cells (Alnajjar et al., 2020). Interestingly, candesartan cilexetil is only effective as the prodrug. Candesartan cilexetil is rapidly ester hydrolyzed in the gastrointestinal tract into the angiotensin II receptor antagonist candesartan. Candesartan was tested in the FRET-based activity assay and found to have no effect. The active agent against the virus is either the intact prodrug or just the cyclohexyl-1-hydroxylethyl carbonate is required. Additionally, to our knowledge, this is the first time the retinoid adapalene has been shown to be effective against SARS-CoV-2.

## Conclusion

The COVID19 pandemic is the worst in the last century and has highlighted the critical need for a rapid response for identifying inhibitors to combat biological outbreaks before they become unmanageable. Leveraging high performance computing, we combined molecular simulations and machine learning to identify compounds that could possibly bind to the selected protein targets. Yet, computational identification of possible compounds is only the first step to finding an inhibitor. The viability of these selected compounds to inhibit protein function is critical and must be tested *in vitro* and *in vivo*. Through experimental binding assay studies between the identified compounds and the selected proteins and virus assays, four compounds (candesartan cilexetil, imatinib, lapatinib, and adapalene) have been shown to inhibit SARS-CoV-2 virus *in vitro*. Interestingly, compounds predicted to bind to the spike protein affected the virus more strongly than the predicted Mpro inhibitors even though the binding site of Mpro is deeper and better defined than the spike binding site. Imatinib, adapalene and candesartan cilexetil had IC50 values of approximately 10 μM against SARS-CoV-2 in an *in vitro* cellular infection assay, but the prodrug candesartan cilexetil shows the most promise as its selectivity index is greater than the limit of the assay.

## Data Availability

Data and results from this study are publicly available at https://covid19drugscreen.llnl.gov.
