# Supplementary material for: Discovery of Small-Molecule Inhibitors of SARS-CoV-2 Proteins Using a Computational and Experimental Pipeline
Source: Front Mol Biosci. 2021 Jul 9;8:678701. doi: 10.3389/fmolb.2021.678701 (PMC8315004; doi:10.3389/fmolb.2021.678701)
Supplement: Supplementary file 1 [file DataSheet1.pdf]

Supplementary Material

1 Supplementary Data

Edmond Y. Lau[1], Oscar A. Negrete[2], W. F. Drew Bennett[1], Brian J. Bennion[1], Monica Borucki[1], Feliza Bourguet[1], Aidan Epstein[3], Magdalena Franco[1], Brooke Harmon[4], Stewart He[3], Derek Jones[3], Hyojin Kim[5], Daniel Kirshner[1], Victoria Lao[1], Jacky Lo[1], Kevin McLoughlin[3], Richard Mosesso[4], Deepa K. Murugesh[1], Edwin A. Saada[4], Brent Segelke[1], Maxwell A. Stefan[4], Garrett A. Stevenson[6], Marisa W. Torres[3], Dina R. Weilhammer[1], Sergio Wong[1], Yue Yang[1], Adam Zemla[3], Xiaohua Zhang[1], Fangqiang Zhu[1], Jonathan E. Allen[3], Felice C. Lightstone[1]*

[1]Lawrence Livermore National Laboratory, Physical Life Sciences Directorate, Biotechnology and Biosciences Division, Livermore, CA, 94550 USA
[2]Sandia National Laboratory, Department of Biotechnologies and Bioengineering, Livermore, CA, 94550 USA
[3]Lawrence Livermore National Laboratory, Computing Directorate, Global Security Computing Division, Livermore, CA, 94550 USA
[4]Sandia National Laboratory, Department Systems Biology, Livermore, CA, 94550 USA
[5]Lawrence Livermore National Laboratory, Computing Directorate, Center for Applied Scientific Computing, Livermore, CA, 94550 USA
[6]Lawrence Livermore National Laboratory, Engineering Directorate, Computational Engineering Division, Livermore, CA, 94550 USA

*** Correspondence:**
Felice Lightstone
lightstone1@llnl.gov

2 Supplementary Figures and Tables

Data and results from this study are publicly available at https://covid19drugscreen.llnl.gov.

Table S1: Total number of simulations used to obtain average MM/GBSA energies for individual compound-protein complexes. The top five docked poses for the individual complex-protein (if all made the minimum energy cutoff) were used for molecular dynamics simulations. Four replicate simulations were performed on the top dock pose and two replicate simulations were performed on the remaining dock poses. The trajectories were pooled to obtain an average MM/GBSA energy for an individual compound-protein complex.

	Drug compounds	Docked poses	Total MD simulations
**Protease1**	914	3810	9446
**Protease2**	934	3875	9618
**Spike1**	134	652	1572
**Spike2**	50	250	600

Table S2: Description of AMPL models used to predict pharmacokinetic and safety properties. All data presented in the manuscript is available at https://covid19drugscreen.llnl.gov.

Description	Dataset size	Public
log solubility	732	
Solubility	732	
log10 human plasma protein fraction unbound	3300	
pIC50 for sodium channel NaV1.5 inhibition	3946	
plasma protein binding fraction unbound, human serum albumin	123759	
log10 rat microsomal clearance rate	30563	
log10 plasma protein binding fraction unbound, human serum albumin	123734	
pIC50 for cytochrome P450 CYP3A4 inhibition	112660	
logD distribution coefficient	27345	
pIC50 for hERG potassium channel inhibition	47251	
log10 human plasma protein binding fraction unbound, alternate model	3019	
log10 rat plasma protein binding fraction unbound	3394	
pIC50 for bile salt export pump inhibition	1149	
log10 human microsomal clearance rate	29162	
log10 steady state volume of distribution in humans	970	public
log10 human microsomal clearance rate, alternate model	1102	public
pIC50 for aurora kinase A inhibition	1791	public
pIC50 for aurora kinase B inhibition	1791	public
pIC50 for muscarinic acetylcholine receptor M1 inhibition	829	public
pIC50 for muscarinic acetylcholine receptor M2 inhibition	535	public

pIC50 for muscarinic acetylcholine receptor M3 inhibition	857	public
pIC50 for histamine receptor H1 inhibition	328	public
pIC50 for Kv1.5 potassium channel inhibition	767	public
pIC50 for sodium channel NaV1.5 inhibition, alternate model	1729	public
log10 steady state volume of distribution in humans, public model	970	public
pIC50 for bile salt export pump inhibition, public model	1120	public
pIC50 for hERG potassium channel inhibition, public model	14023	public
mm/gbsa for spike1	186235	public
mm/gbsa for protease2	195379	public
mm/gbsa for protease1	194814	public
pIC50 for caspase 1 inhibition	598	public
pIC50 for caspase 3 inhibition	2063	public
pIC50 for cathepsin K inhibition	1506	public
pIC50 for cathepsin L1 inhibition	1633	public
pIC50 for cytochrome p450 3A4 inhibition, public model	6515	public
pIC50 for hERG potassium channel inhibition, public model 2	11920	public
qualitative estimate of druglikeness	rdkit function	public
synthetic accessibility score	rdkit function	public

Table S3: Inhibition of pseudovirus by computationally selected spike binding compounds.

Compound	%Infection		%Inhibition	%Inhibition
Name	AVG	Stdev	AVG	No NegValues
Phenolphthalein	119.0	18.4	-19.0	0.0
Novobiocin Sodium	117.4	35.9	-17.4	0.0
Prednisone	126.6	19.7	-26.6	0.0
Ketanserin	142.6	29.4	-42.6	0.0
Chlortalidone	118.1	16.4	-18.1	0.0
Adapalene	104.8	29.0	-4.8	0.0
Bendroflumethiazide	134.2	38.4	-34.2	0.0
Olaparib	128.2	33.0	-28.2	0.0
Fluorescein	536.3	242.3	-436.3	0.0
Ethynylestradiol	127.7	44.8	-27.7	0.0
Olmesartan Medoxomil	104.0	22.8	-4.0	0.0
Nilotinib	83.3	29.0	16.7	16.7
Darifenacin HBr	96.9	41.8	3.1	3.1
Lumacaftor	121.3	22.8	-21.3	0.0
Mestranol	147.6	45.6	-47.6	0.0
Dutasteride	61.3	18.9	38.7	38.7
Ciclesonide	184.6	26.1	-84.6	0.0
Etravirine	99.3	17.4	0.7	0.7
Pazopanib	94.0	10.2	6.0	6.0
Rilpivirine	73.3	34.7	26.7	26.7
Lapatinib Ditosylate	55.4	14.3	44.6	44.6
Imatinib	8.6	6.0	91.4	91.4
Mizolastine	103.5	7.4	-3.5	0.0
Raltegravir	119.6	38.6	-19.6	0.0
Ivacaftor	228.8	18.5	-128.8	0.0
Nalmefene hydrochloride	104.0	24.0	-4.0	0.0
Troglitazone	103.0	28.0	-3.0	0.0
Zafirlukast	110.5	25.1	-10.5	0.0
Icotinib	130.2	16.8	-30.2	0.0
Vorapaxar	143.0	50.2	-43.0	0.0
Ajmaline	90.6	9.3	9.4	9.4
Gestrinone	90.2	12.1	9.8	9.8
Trametinib DMSO solvate	97.6	17.4	2.4	2.4
Tasosartan	115.2	26.9	-15.2	0.0
Ace2-Fc	8.0	3.4	92.0	92.0
NoTreatment	100.0	1.9	0.0	0.0

2.1 Supplementary Figures

Figure S1:

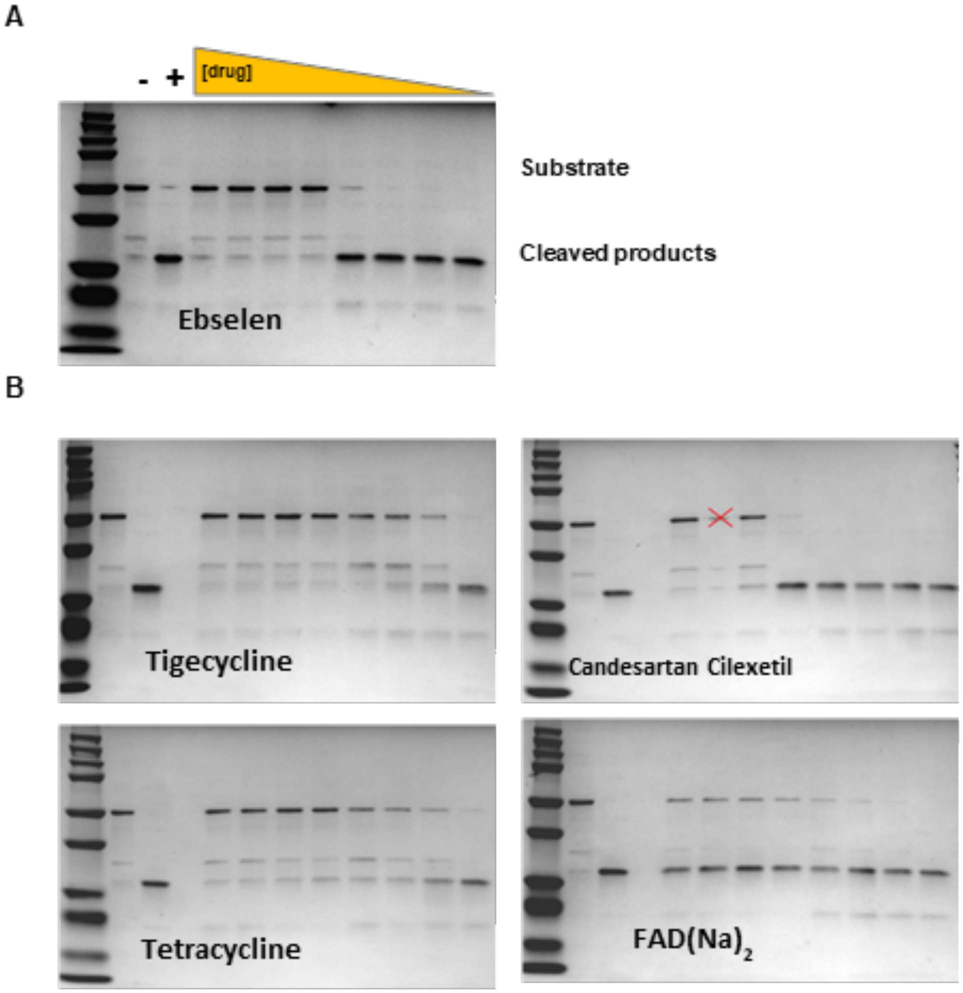

Figure S1. Gel electrophoresis analysis used for densitometry-based normalization of M^{pro} inhibitor IC_{50} data. (A) Ebselen was used as a control M^{pro} inhibitor against the FRET-based substrate and the cleavage products from the IC_{50} studies shown in Figure 1D were analyzed by gel electrophoresis and Coomassie staining. (B) The M^{pro} inhibitor hits were also analyzed similarly to Ebselen. Negative (-) controls indicate no protease conditions and positive (+) controls indicate no drug treatment conditions. Decreasing concentrations of inhibitors are shown from left-to-right on the gels.

Table S4: Computationally selected M^{pro} binders ability to inhibit in FRET-based activity assay.

Screen ID	Compound name	Normalized Percent FRET inhibition
1	Pyronaridine	-8.326469859

2	Acarbose	-3.443413545
3	Alvimopan	-3.261275118
4	Ethynyl estradiol	-3.167551476
5	Imatinib	-2.881543041
6	Hyaluronic acid	-2.844182585
7	Maraviroc	-2.689853634
8	Rupatadine Fumarate	-2.414611759
9	Dutasteride	-2.375936492
10	Irinotecan hydrochloride trihydrate	-2.220367155
11	Azilsartan Medoxomil	-1.990101712
12	Atazanavir	-1.967080129
13	Tasosartan	-1.956784917
14	Ibrutinib	-1.908657901
15	Darunavir Ethanolate	-1.838476805
16	Ketoconazole	-1.797246341
17	Risperidal	-1.672488216
18	Lapatinib Ditosylate	-1.574075912
19	Periplocin	-1.535996031
20	Fluocinonide	-1.512130985
21	Ezetimibe	-1.341180848
22	Saquinavir	-1.335400645
23	Cefmenoxime hydrochloride	-1.303795584
24	Efonidipine	-1.161647234
25	Mestranol	-1.101041925
26	Pazopanib	-0.95532126
27	Ouabain octahydrate	-0.947829323
28	Ivermectin	-0.915827338
29	Azlocillin sodium	-0.914760605
30	MALTOTETRAOSE	-0.905532126
31	Etravirine	-0.894666336
32	Isavuconazole	-0.885661126
33	Olmutinib	-0.794889606
34	Ginkgolide C	-0.741850657
35	Icotinib	-0.627933515
36	Rutin	-0.553634334
37	Tacrolimus	-0.416745225
38	Vorapaxar	-0.416199454
39	Sofosbuvir	-0.399751923
40	Ciclesonide	-0.393053833
41	Diosmin	-0.377797073
42	Etoposide	-0.323666584
43	Mizolastine	-0.317166956

44	Cefoperazone	-0.306673282
45	Lomerizine hydrochloride	-0.276531878
46	Dasatinib	-0.250409328
47	Novobiocin Sodium	-0.227809477
48	Regadenoson	-0.187670553
49	Fluocinolone acetonide	0.024906971
50	Digitoxin	0.196899032
51	Adapalene	0.235921608
52	Cabozantinib Malate	0.386306128
53	Vinorelbine	0.454056065
54	Paritaprevir	0.757727611
55	Doxazosin mesylate	0.918977921
56	Trametinib DMSO solvate	1.515951377
57	Eptifibatide acetate	2.0560903
58	Cefotetan	2.140163731
59	Olmesartan Medoxomil	2.830166212
60	Teniposide	3.019498884
61	Fosinopril sodium	3.314735798
62	Aprepitant	3.36641032
63	Calcium N5-methyltetrahydrofolate	3.743115852
64	Zafirlukast	3.826643513
65	Enoxolone	4.005656165
66	Vecuronium bromide	4.129297941
67	Ginsenoside Rg1	4.192259985
68	Rilpivirine	5.867228975
69	Brigatinib	5.965765319
70	ABT199	6.272661871
71	Nilotinib	6.659141652
72	Cefpiramide acid	7.372587447
73	Azelnidipine	8.169957827
74	Linagliptin	8.281716696
75	Pancuronium bromide	10.01384272
76	Rocuronium bromide	11.30109154
77	Bendroflumethiazide	11.621161
78	Hydroxyzine Pamoate	12.92944679
79	Calcium Levofolinate	13.00131481
80	C10 (control)	13.05065741
81	Argatroban	13.45514761
82	Cefoperazone sodium	14.80501116
83	Lopinavir	18.16271397
84	Eltrombopag Olamine	23.71875465
85	Nelfinavir Mesylate	30.75008683

86	Tetracycline	40.29213595
87	Tigecycline	56.53225006
88	Simeprevir	72.89630365
89	EBSELEN (control)	117.2592409
90	Flavin Adenine Dinucleotide Disodium	148.8484247
91	Candesartan Cilexetil	152.7055321
	No drug	0
	Protease only	100

Italicized are controls
